# Metabolite profiling of the ripening of Mangoes *Mangifera indica* L. cv. ‘Tommy Atkins’ by real-time measurement of volatile organic compounds

**DOI:** 10.1007/s11306-016-0973-1

**Published:** 2016-02-18

**Authors:** Iain R. White, Robert S. Blake, Andrew J. Taylor, Paul S. Monks

**Affiliations:** Department of Chemistry, University of Leicester, Leicester, LE1 7RH UK; Flavometrix Ltd., Sutton Bonington, Loughborough, Leicestershire LE12 5RD UK

**Keywords:** *Mangifera indica*, Tommy Atkins, PTR–ToF–MS, VOCs, Ripening, Mango

## Abstract

Real-time profiling of mango ripening based on proton transfer reaction-time of flight-mass spectrometry (PTR–ToF–MS) of small molecular weight volatile organic compounds (VOCs), is demonstrated using headspace measurements of ‘Tommy Atkins’ mangoes. VOC metabolites produced during the ripening process were sampled directly, which enabled simultaneous and rapid detection of a wide range of compounds. Headspace measurements of ‘Keitt’ mangoes were also conducted for comparison. A principle component analysis of the results indicated that several mass channels were not only key to the ripening process but could also be used to distinguish between mango cultivars. The identities of 22 of these channels, tentatively speciated using contemporaneous GC–MS measurements of sorbent tubes, are rationalized through examination of the biochemical pathways that produce volatile flavour components. Results are discussed with relevance to the potential of headspace analysers and electronic noses in future fruit ripening and quality studies.

## Introduction

According to the Food and Agriculture Organization (FAO) database on global crop statistics (2014), the worldwide production of mangoes has almost quadrupled since 1961 with an exponential increase of 2.6 % years^−1^ to 42 Mt in 2012. This makes mango one of the most important and popular tropical fruits. India produces approximately 40 % of the world’s supply and although there are more than a thousand varieties of Indian mango, only 30 are grown on a large scale for distribution across the subcontinent (Jha et al. 2010). In the West, whilst there is a growing demand for more variety, the five major mango cultivars are ‘Ataulfo’, ‘Haden’, ‘Kent’, ‘Keitt’ and ‘Tommy Atkins’; the latter dominating the world export trade owing to its durability and long shelf-life. Generally mangoes are harvested at the mature green stage i.e., when physiologically mature but before the characteristic rise in respiration activity and phytohormone production associated with climacteric fruit (Lakshminarayana et al. 1970). Imported mangoes are generally ripened to eating quality by the wholesaler, retailer or consumer. In general, the time taken to reach a state that is of desirable taste and texture is approximately 8–12 days, however, this is highly dependent on variety, prevailing environmental conditions and harvest maturity (Lakshminarayana 1980). During the course of this ‘ripening period’, the colour, firmness, size, shape and aroma of the fruit changes. These factors, alongside total soluble solid content, acidity, physiological weight, defects, pulp composition and moisture content, can be used to assess the quality and maturity of mango fruit (Jha et al. 2010). Knowledge of specific ripeness stage is necessary in order to assess optimal harvest maturity and the quality of fruit as it is marketed to the consumer as well as to evaluate postharvest packaging and handling strategies.

Measuring chemical properties to assess fruit ripening is advantageous as they can be directly linked to eating quality. However, previous methods were often invasive, requiring the fruit to be pulped before chemical analyses. More recently, non-destructive methods have been developed to assess fruit quality such as those based on near infrared spectroscopy (Saranwong et al. 2004) and electronic noses (Röck et al. 2008). Electronic noses and headspace aroma detectors measure the volatile compounds that contribute to fruit flavour. Electronic noses, based on metal oxide sensors, do not identify individual species but rather, record signals as unique patterns which can then be retrospectively related to a specific process or application; including environmental control (Keller et al. 1995), medical diagnostics (Turner and Magan 2004) and food applications (Schaller et al. 1998). Lebrun et al. (2008) used an electronic nose to measure differences between the aroma of ‘Cogshall’, ‘Kent’ and ‘Keitt’ mango cultivars and Jha et al. (2010) reviewed the potential that these and other non-destructive detectors have in measuring mango quality.

Volatile aroma compounds from mango headspace have also been measured by gas chromatography mass–spectrometry (GC–MS) by utilising solid-phase-micro-extraction (SPME) (Pawliszyn 1997). SPME is solvent-free, rapid and sensitive and has been used to analyse the volatile aroma compounds from both pulped (Shang et al. 2002) and intact mango (Lalel et al. 2003). Whilst GC–MS based methods such as these are capable of identifying individual species within complex gaseous mixtures, the sampling and analytical time required can render analyses intensive whilst methods specific to lightweight oxygenated VOCs cannot analyse heavier aliphatic aroma compounds. In the present study, a direct mass spectrometer-based technique (chemical ionisation reaction (CIR) mass spectrometry) is employed in real-time to observe the VOCs emitted during the mango ripening process in a non-destructive manner.

The proton transfer reaction mass spectrometry (PTR–MS) technique, pioneered by Lindinger et al. (1998), has become a powerful research tool for investigating the composition of gas-phase mixtures for a variety of applications (Blake et al. 2009). CIR-MS expanded PTR–MS to other reagent ions such as NO^+^ and O_2_^+^ (Blake et al. 2006). PTR ionisation proceeds by proton donation, precluding the need for sample separation due to its selectivity and soft ionization, enabling measurements of the ripening process to be acquired continuously in a fully automated sequence. Direct MS devices such as those which are based upon PTR ionisation [including PTR–MS, PIT–MS (Steeghs et al. 2007) and SIFT-MS (Smith and Spanel 2005)] have been deployed extensively in studies of the behaviour of organic material and foodstuff including fruit ripeness and quality (Ruth et al. 2008; Vezzaro et al. 2011) and based upon spectral fingerprinting (e.g., Biasioli et al. 2006 in work on strawberry cultivars). Capellin et al. (2012) described the potential of PTR–ToF–MS coupled with multivariate and data mining methods and demonstrated the use of this technique by examining varietal and clonal apple VOC fingerprints. Recently, Taiti et al. (2015) used the PTR–ToF–MS technique to investigate how different shipping systems affect ‘Kent’ mango quality by measuring volatile compounds alongside physicochemical properties such as firmness and pH. Furthermore, in a separate study (Taiti et al. 2015), traditional methods of ripening observation were compared to chemical analyses of pulps of several fruits. Taiti et al. found that by pooling the entire dataset together, the two different ripeness stages under study could be well constrained (not possible using data for fruit firmness and skin colour alone) and suggested that certain compounds may have stronger predictive powers than others.

Few studies have reported the dynamics of volatile organic compound (VOC) production during mango ripening. This information could be important in the development of a non-destructive method of assessing ripeness which could have wide applications in research and commerce. Here we report observations on mango ripening using the ‘Tommy Atkins’ cultivar with subsidiary work taken on the ‘Keitt’ cultivar for comparison. PTR Time-of Flight (ToF) MS headspace measurements were acquired over several 100 hours. Headspace samples were also taken on sorbent tubes at discrete intervals and processed by an automated thermal desorption (ATD) GC–MS to identify the volatile compounds. The results are rationalised following observation of the changes in the relative concentration of emitted species by relating the biogenesis of aroma volatiles to physiological changes within the fruit.

## Experimental

### General experimental procedure

Ripeness stages defined in this study were based upon subjective assessments of fruit colour, as in Shorter and Joyce (1998) i.e., Stages I–V represent complete green coverage to complete yellow coverage respectively (Shorter and Joyce 1998). Two ‘Tommy Atkins’ experiments were conducted based on triplicate PTR–MS analyses of individual stage II (75 % green) mangoes. The first mango was analysed under a dry nitrogen atmosphere whilst the second headspace analysis was performed using VOC-scrubbed ambient air in a temperature controlled (T = 23 °C) environment. Prior to analysis, mangoes were checked for evidence of defects and symptoms of disease. A third ‘Tommy Atkins’ experiment was conducted in order to give an indication of reproducibility as well as to assess the suitability of the technique for use in batch monitoring; in this experiment the headspace above a chamber containing five mangoes was analysed using VOC-scrubbed ambient air. This experiment was monitored continuously over a 444 h period in order to track the ripening process across different ripeness stages. In all experiments, headspace air was sampled at a flow rate of 120 mL/min for either direct analysis by PTR–ToF–MS or trapping onto sorbent tubes for analysis by ATD–GC–MS. For comparison, two experiments were conducted using ‘Keitt’ mangoes. The first involved headspace analysis of an individual stage II ripened mango using VOC scrubbed air whilst the second ‘batch’ experiment analysed air sampled above 5 ripening mangoes at stages, I, III and V (with a total interval between ripeness stages lasting approximately 451 h). All headspace analyses of ‘Keitt’ mangoes were performed in triplicate. Background signals were determined for each mass channel based on analyses of N_2_ (for the first experiment) or VOC-scrubbed ambient air (for all other experiments) conducted both prior to fruit analysis and periodically during the experiments.

The experimental set-up and principle of the PTR–ToF–MS technique is described elsewhere (see Blake et al. 2004; Wyche et al. 2007). In general, the mass spectrometer (based on a KORE model P-4500-A design (KORE Technologies, Ely, UK) with extensive in-house modifications) uses α-particles generated from an ^241^Am source to create positively charged reagent ions from a fixed flow of reagent gas. In the present study, H_3_O^+^ (hydronium) ions were produced by bubbling pure nitrogen gas through de-ionised water at a flow rate of 32 ml/min. The detector cycle time, equivalent to the flight time of an ion equal to approximately 300 *m/z*, was integrated over a period of 300 s. Ionisation conditions were equivalent to an average electric field to number density ratio (E/N) of approximately 80 Td over the main body of the drift tube, with a short collision-induced-dissociation (CID) region at the end of the drift tube equal to 170 Td. PTR fragmentation patterns were obtained by analyzing standard samples prepared by either gravimetric dilution of pure chemicals into a multi-component mixture suspended in nitrogen (Linde Specialty Gases Ltd, UK) or by in-house volumetric dilution of VOC/hexane mixtures into 10 L Tedlar bags (SKC ltd.) containing dry nitrogen.

The ATD–GC–MS system consisted of a Markes UNITY series 2 thermal desorber (Markes International, UK) linked to an Agilent 7890A gas chromatograph with a 5975C mass selective detector (Agilent Technologies, Ltd.). Samples were collected for 4 min onto sorbent tubes packed with approximately 300 mg Tenax TA 35/60 and Carbograph 1TD 40/60. Samples were desorbed at 280 °C, 40 mL/min He flow (20 mL/min to split vent) for 3 min and refocused on a general purpose cold trap (U-T11GPC-2S, Markes Intl., UK) held at −10 °C. The trap was then rapidly heated and held at 300 °C with 10 ml/min split flow as analyte desorbed onto a deactivated silica transfer line at 150 °C. Volatile components were then transferred to the GC column (30 m × 0.25 mm i.d. × 0.25 μm DB-5MS capillary column, Agilent J&W, Santa Clara, CA) with 1.6 ml/min He carrier gas flow. The GC oven conditions were: 60 °C for 3 min, 2 °C/min to 100 °C, 15 °C/min to 300 °C, hold for 5 min. The MS, fitted with an EI source, operated at 70 eV with a source temperature of 230 °C and a quadrupole temperature of 150 °C. Mass spectra were scanned over a range *m/z* 35–550.

Table 1 lists compounds identified by ATD–GC–MS based on: forward and reverse spectral match values obtained following sample tube analysis; comparison of temperature programmed retention indices (TPRIs) with reference values; and the analysis of pure headspace samples trapped onto sorbent tubes, analysed in the same manner as mango headspace samples. Retention indices were determined following the sorption and analysis of pure n-alkane standards (C_5_–C_16_) and are presented in Table 1 alongside values determined by Babushok et al. (2011) from a data distribution analysis of frequently reported plant essential oils compiled for temperature programmed conditions typically used in GC measurements. PTR fragmentation patterns obtained through the analysis of standard mixtures are also provided in Table 1. Pure chemicals were obtained from Sigma-Aldrich (Poole, Dorset, UK).

**Table 1:** Species identity for compounds analysed in ‘Tommy Atkins’ mango headspace samples based upon forward and reverse match hits and temperature programmed retention indices (TPRI), verified with (i) standard samples run on CIR-MS and (ii) standard samples run on GCMS

PTR signal *(m*/*z)*	(i)	Compound	Forward match	Reverse match	TPRI	Ref. RI	(ii)
15, 33	•	Methanol	–	–	–	–	
45	•	Acetaldehyde	–	–	–	–	
29, 47	•	Ethanol	–	–	–	–	
59	•	Acetone	–	–	–	–	
43, 61	•	Propanol	819	850	569	568 (Pino et al. 2005)	
73		Methyl ethyl ketone	802	802	583	583 (Miyazaki et al. 2011)	
43, 61, 89		Ethyl acetate	958	958	600	611	
57, 75	•	Butanol	823	826	654	668 (Pino et al. 2005)	
43, 103		Ethyl propanoate	945	950	708	714 (Pino et al. 2005)	
43, 103	•	Methyl butanoate	928	929	715	724 (Pino et al. 2005)	
71, 89	•	Pentanol	802	802	757	767 (Pino et al. 2005)	
43, 57, 71, 89, 117		Ethyl isobutanoate	961	962	746	755	
(fragmentation not measured) 115		Methyl crotonate	926	926	751	756 (Pino et al. 2005)	
43, 57, 71, 89, 117	•	Ethyl butanoate	930	930	797	799	•
(fragmentation not measured) 101		Ethyl crotonate	908	909	834	835 (Pino et al. 2005)	•
43, 57, 71, 89, 117		2-Methyl ethyl butanoate	935	950	840	842 (Pino et al. 2005)	•
(fragmentation not measured) 115		Methyl tiglate	802	854	858	860 (Costa et al. 2008)	
103, 131		Propyl butanoate	949	956	897	896	•
103, 131		Ethyl pentanoate	893	893	901	901	
43, 57,71, 99, 131	•	Methyl hexanoate	931	935	922	924	
81, 95, 137	•	α-Pinene	954	954	930	936	•
(fragmentation not measured) 133		Butyl-3-hydroxy ethyl ester	816	816	932	937 (Fan et al. 2009)	
(fragmentation not measured) 129		Ethyl tiglate	935	936	934	938 (Costa et al. 2008)	
43, 71, 99, 117, 145		Isobutyl butanoate	878	878	950	955 (Robinson et al. 2012)	
81, 95, 137		β-Pinene	852	862	979	978	
81, 95, 137		β-Myrcene	909	909	990	989	•
43, 71, 99, 117, 145	•	Butyl butanoate	925	930	998	994 (Pino et al. 2005)	•
43, 71, 99, 117, 145	•	Ethyl hexanoate	954	954	1001	1000	
81, 95, 137	•	3-Carene	929	944	1008	1011	•
(fragmentation not measured) 135		m-Cymene	837	908	1020	1022	
(fragmentation not measured) 135		p-Cymene	913	913	1022	1024	
81, 95, 137	•	d-Limonene	907	909	1025	1030	•
81, 95, 137		β-Ocimene	888	888	1046	1048	
81, 95, 137	•	Terpinolene	907	920	1087	1087	•
43, 57, 117, 159		Propyl hexanoate	921	921	1097	1096 (Robinson et al. 2012)	
43, 57, 117, 159	•	Ethyl heptanoate	895	895	1101	1098 (Robinson et al. 2012)	•
57, 159	•	Methyl octanoate	935	946	1125	1128	
43, 57, 71, 145, 173		Butyl hexanoate	918	918	1196	1188 (Pino et al. 201)	•
43, 57, 71, 145, 173	•	Ethyl octanoate	934	935	1199	1196	•
43, 57, 155, 187	•	Methyl decanoate	906	909	1316	1326	
81, 109, 121,135, 137, 149, 205		α-Cubebene	865	865	1349	1351	
81, 109, 121,135, 137, 149, 206		α-Ylangene	835	838	1375	1370	
81, 109, 121,135, 137, 149, 205		α-Copaene	953	962	1378	1376	
81, 109, 121, 135, 137, 149, 205		β-Elemene	894	896	1391	1390	
81, 109, 121, 135, 137, 149, 205		β-Cubebene	842	850	1392	1387	
43, 57, 71, 155, 201	•	Ethyl decanoate	908	908	1406	1395	
81, 109, 121,135, 137, 149, 205		α-Gurjinene	917	918	1409	1409	
81, 109, 121,135, 137, 149, 205		α-Cedrene	804	858	1415	1412	
81, 109, 121,135, 137, 149, 205	•	β-Caryophyllene	904	904	1420	1420	•
81, 109, 121,135, 137, 149, 205		Bicyclosesquiphellandrene	865	865	1468	1464	
81, 109, 121,135, 137, 149, 205		γ-Muurolene	884	884	1482	1476	
81, 109, 121, 135, 137, 149, 205		Germacrene D	898	944	1486	1481	
81, 109, 121, 135, 137, 149, 205		α-Muurolene	883	883	1499	1498	
(fragmentation not measured for C_14_ ester)229		Ethyl dodecanoate	882	907	1607	1593	•
81, 109, 121,135, 137, 149, 205		γ-cadinene	905	905	1520	1513	
81, 109, 121,135, 137, 149, 205		λ-Cadinene	915	915	1530	1523	
(fragmentation not measured for C_15_ ester)257		Ethyl tetradecanoate	824	824	1793	1794	

TPRIs are presented next to reference retention indices from Babushok et al. (2011) unless otherwise stated

## Results and discussion

**Fig. 1:**
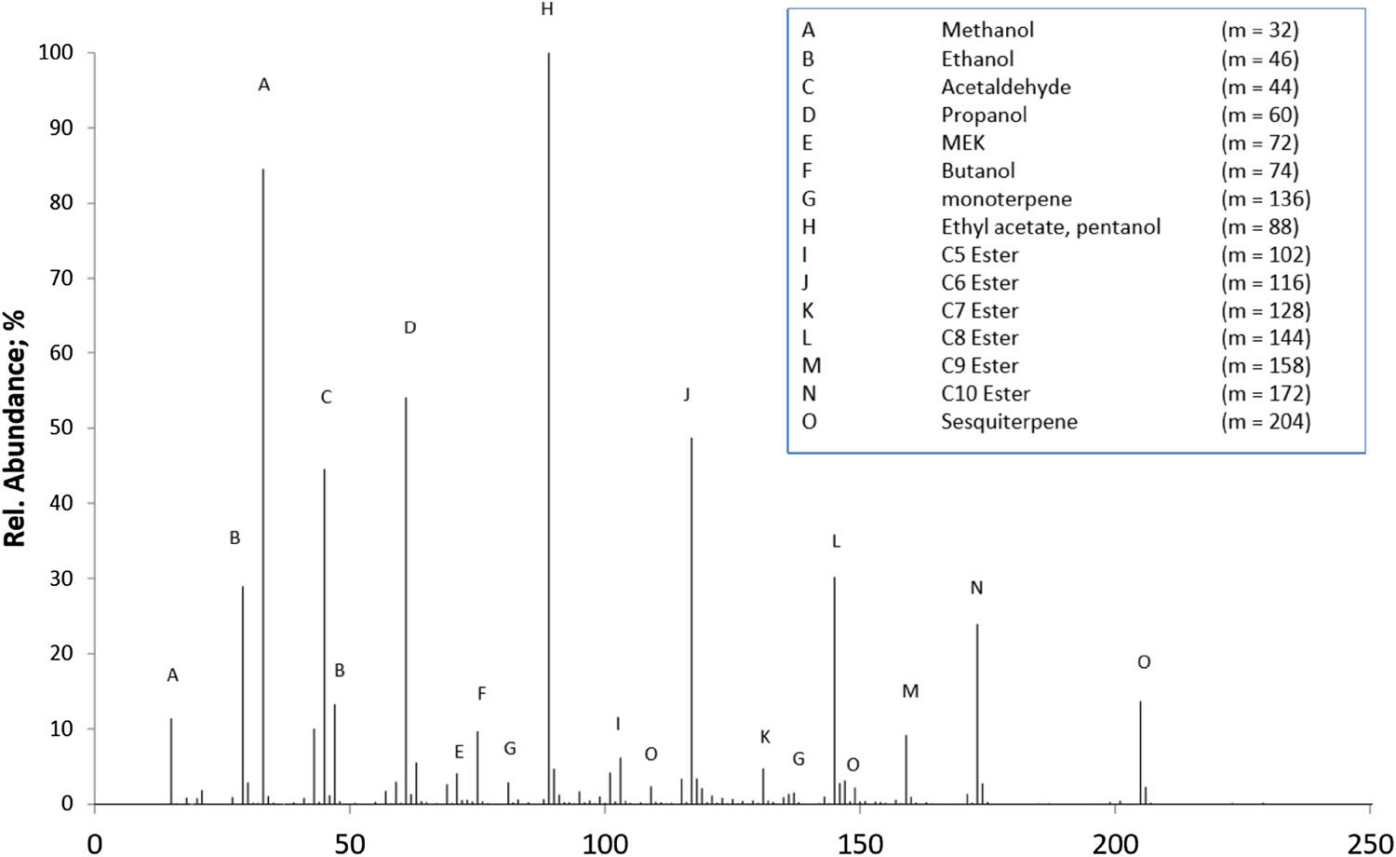
PTR mass spectrum taken during the batch ‘Tommy Atkins’ analysis at ripeness stage IV (non-deconvoluted spectrum—peak labeling based on protonated parent ions)

**Fig. 2:**
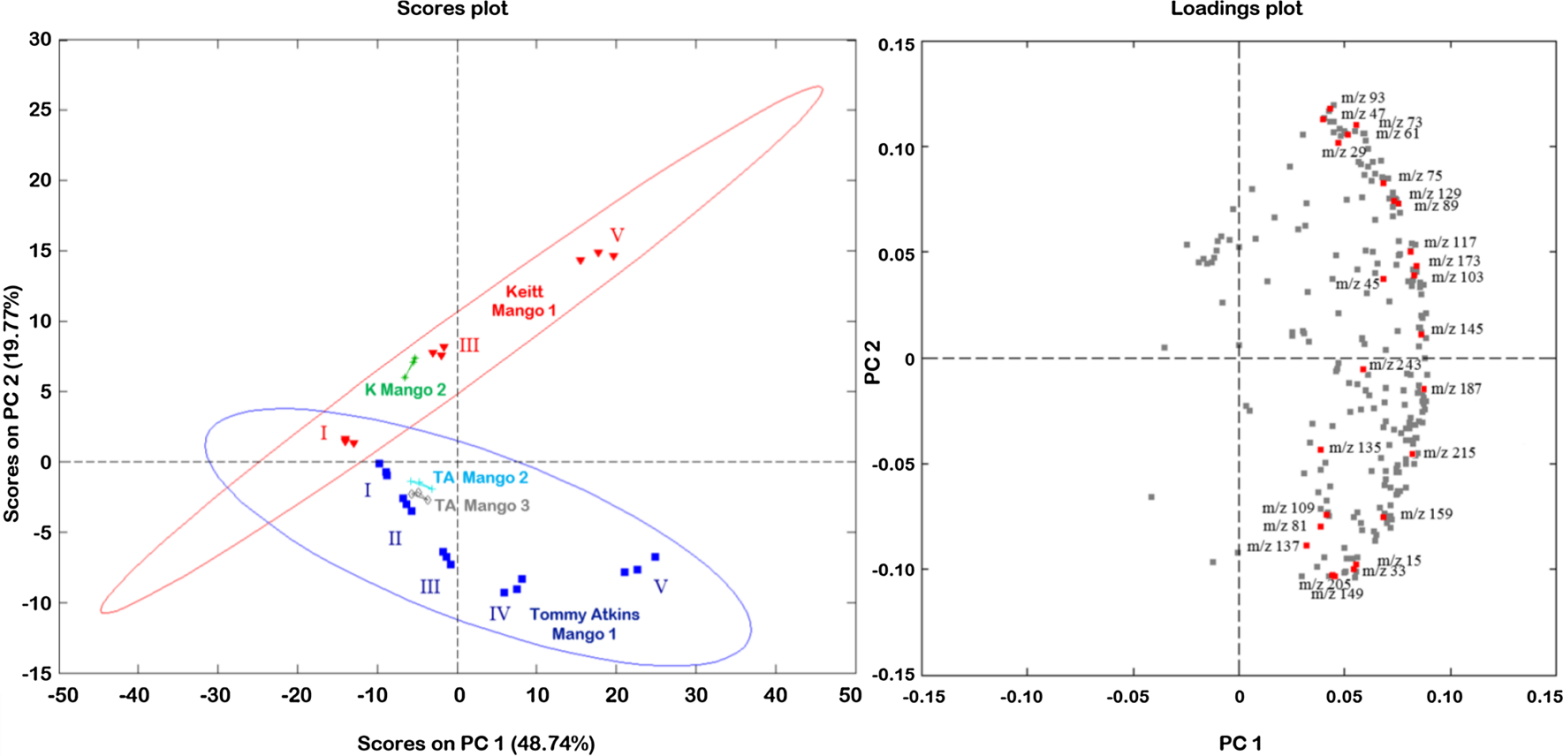
Principle component analysis of the mango ripening experiment (*ellipses* represent a 95 % confidence interval). Tommy Atkins Mango 1 = batch experiment, TA Mangos 2 and 3 represent single stage II mangos analyzed under dry nitrogen and VOC scrubbed air, respectively. Keitt Mango 1 = batch experiment, K Mango 2 represents the single stage II Keitt Mango experiment

Headspace measurements with the PTR–ToF–MS provided an extensive dataset on the characteristic emissions of the ripening ‘Tommy Atkins’ mangoes. Figure 1 shows a typical PTR Mass Spectrum taken during the headspace analysis of a ripening stage IV Tommy Atkins Mango. In order to identify which of the signals presented in Fig. 1 correspond to compounds important to the ripening process, as well as those that represent the aroma compounds specific to fruit variety, a principle component analysis (PCA) was conducted using PLSToolbox (version 6.21, EVRI ltd.) on a MATLAB platform by normalizing all values to the hydronium signal at m/z = 19 (equal to 10^6^ counts of normalized hydronium) before background subtraction and autoscaling. In total, 33 samples and 248 m/z values were computed by PCA. Figure 2 shows the results from the PCA model whereby ‘Tommy Atkins’ can clearly be distinguished from ‘Keitt’ based on the scores plot for PCs 1 and 2. Furthermore, PCA highlighted the main differences between the samples across the different maturation stages.

### Batch versus single experiment

Several key observations can be made from a qualitative assessment of Fig. 2: firstly, that mangoes can be discriminated based on their degree of maturation. On observation of the scores plot, all of the stage II mangoes appear to approximately align on the x axis. Furthermore, based on the apparent variation within each triplicate analysis, the different experimental conditions (i.e., conducting the experiment in a dry nitrogen atmosphere (TA Mango 2) versus scrubbed ambient air (TA Mango 3)) appears to have made a negligible difference to emission patterns. Fruit variety also appears to be separated on the scores plot, the extent of which becomes more exaggerated as the fruits ripen. Scores from the batch experiments (TA mango 1 and Keitt mango 1) seem to overlay the scores from the single experiments (TA2, TA3 and K mango 2), well within the 95 % confidence ellipses. Based on this result, in order to rationalize mass channel assignment and interpret results, it is assumed that batch and single fruit ripening varies only in speed of maturation due to an accumulation of important phytohormones and compounds that stimulate ripening, and do not vary in the relative levels of components that give rise to a fruit cultivar’s distinctive and distinguishable aroma.

Inspection of the loadings presented in Fig. 2 may potentially provide information on key compounds that determine fruit variety and ripening: for example, ions 93, 73, 61, 47, and 29 appear to be associated with Keitt mangoes. In this case, the location of a loading on a biplot may be influenced by unknown fragments and isomers and thus ascribing a single species identity to an individual loading remains tentative. For example, ions such as 61, 47, and 29 are likely associated with alcohols and their fragments, such as ethanol and propanol, however 61 is also a known fragment of ethyl acetate; 73 likely represents a C_4_ aldehyde or ketone, and while 93 could represent toluene, it is also a known fragment of the essential oil cymene (Taiti et al. 2015). Toward the bottom of the loadings plot, ions such as 205, 149, 33 and 15 appear to be associated with Tommy Atkins ripening, the first two likely represent sesquiterpene flavour components, while the latter are known fragments of methanol. Finally, several masses appear to load onto PC1 and their emission is therefore likely indicative of fruit ripening, for example, ions 243, 187 and 145 are known fragments and parent ions of esters (Aprea et al. 2007).

**Fig. 3:**
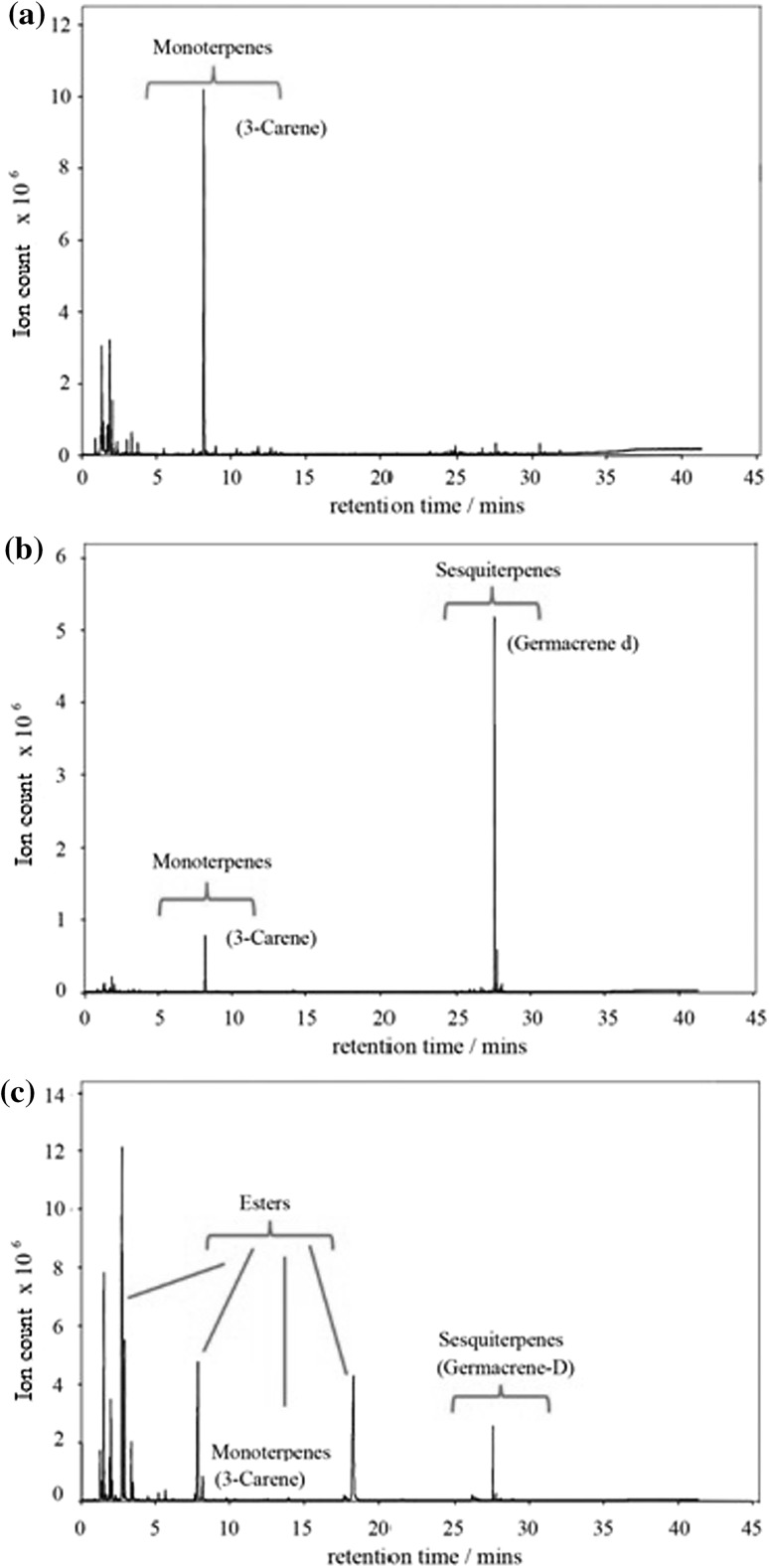
ATD-GC–MS Analysis during the ripening of the batch ‘Tommy Atkins’ experiment at **a** 48 h, **b** 96 h and, **c** 192 h

**Fig. 4:**
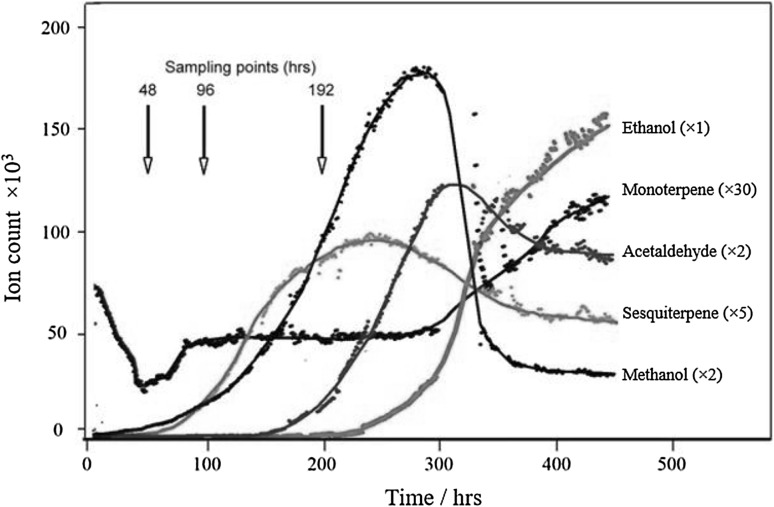
Time profiles for selected species (in ncps) during the ‘batch’ ‘Tomy Atkins’ analysis (the series have been magnified as a visual aid by the factor shown in *brackets*)

Highlighted in the loadings plot for Fig. 2 are a small but significant group of mass channels that appear to not only have a significant role in constraining both ripening stage and fruit variety, but can also be attributed to well known lightweight VOC metabolites which are established as important to the ripening process. In order to support the identification of these mass channels and thus speciate the mass spectrum displayed in Fig. 1, selected samples were analysed by GC–MS. Figure 3 shows the most abundant compounds analysed by ATD–GC–MS which have been used to assign the ions labelled in Fig. 1. Key volatile components comprise hydrocarbons, aldehydes, ketones, alcohols and esters. In total, 57 compounds were tentatively identified (Table 1) following deconvolution of PTR mass spectra based upon their known sensitivities and, where applicable, PTR fragmentation patterns. For situations where several isobars may be detected in a single PTR mass channel, GC–MS data can help to identify species in an unambiguous manner (Lindinger et al. 2005; Pozo-Bayon et al. 2008; Taylor et al. 2003). In this case, GC–MS data provided speciation through comparison of EI fragmentation patterns with the NIST MS reference database verified by their unique TPRIs. Through comparison of PTR–MS and GC–MS analyses, the loadings highlighted in Fig. 2 were tentatively identified, the time series for which are displayed in Fig. 4 showing how the response from these selected compounds varied over the ‘batch’ ‘Tommy Atkins’ experiment.

Based on these results the PCA model has proven a useful tool to identify some of the most important mass channels regarding mango ripening. Furthermore, these results suggest that despite the large number of previously identified flavour components and the far-reaching analytical capability of the PTR–ToF–MS technique, mango fruits (and in particular their ripeness stage) can be characterized by a small number of signals that represent well known lightweight VOCs that are both important and ubiquitous metabolites. In studies such as this, where the number of data points exceeds sample size, there is a risk of drawing conclusions from voodoo correlations (Miekisch et al. 2012) and so in the following sections the selection and identities of these metabolites are rationalized through reference to their biosynthetic production.

### Rationalization of m/z selection and assignment

#### Monoterpenes and Sesquiterpenes (m/z 137, 81, 205, 109, 121 and 149)

The biosynthesis of terpenoids is often restricted to specific tissues at their sites of utilization; they comprise phytoalexins and are an important class of volatile aroma compounds found in fruits. Monoterpenes have a wide variety of scent and potentially their autooxidation following production may give rise to many other odiferous compounds (Grayson 2000). The change in the monoterpene signal in Fig. 4 (*m/z* = 137, 81) exhibits a complex structure: this compound class was emitted strongly initially and dropped off noticeably in the first 50 h of observation. The signal plateaus at approximately 100 h before the terpene concentration increased significantly towards the end of the experiment. Lalel et al. (2003) found the most abundant monoterpene to be α-terpinolene, consistent with previous ‘Kensington Pride’ analyses (Bartley and Schwede 1987; MacLeod et al. 1988). The most abundant monoterpene measured in the present study was 3-carene for the ‘Tommy Atkins’ cultivar (the second most abundant terpene measured by Lalel et al. for ‘Kensington Pride’ (Lalel et al. 2003) which was found to exhibit a very similar profile to that observed here up to 4 days). MacLeod and Snyder (1985) found that 3-carene was the most abundant monoterpene in the pulp of ripe ‘Tommy Atkins’ and, along with Olle et al. (1997), ‘Keitt’ mangoes. In order of contribution to total peak area, the monoterpenes identified by GC–MS analysis include 3-Carene (93 %), d-limonene (2 %), terpinolene (2 %), α-pinene (1 %), β-pinene (<1 %), and β-myrcene (1 %) (based on the relative peak area detected by GC–MS). Taiti et al. (2015) suggested that ions 137 and 81, attributed to monoterpenes, and ion 45 held the most discriminating power during a study into fruit ripening following the analysis of mango pulp.

The interplay between mono and sesqui-terpene levels is complex and varies markedly between cultivars. Monoterpenes and sesquiterpenes are produced by the enzymatic conversion of geranyl disphosphate (GDP) and farnesyl diphosphate (FDP) by terpene synthases. GDP and FDP are biosynthesised by prenyl transferases (GDP and FDP synthase) from the basic terpenoid building blocks, isopentyl phosphate (IPP) and dimethyl allyl diphosphate (DMAPP). Whilst both synthetic pathways involve sequential C_5_-isoprenoid unit addition forming prenyl diphosphates, monoterpene synthesis is currently thought to proceed via formation of IPP and DMAPP following the non-mevalonate (MEP) pathway in the plastids. Conversely, it is generally considered that the building blocks for sesquiterpene formation are formed through the mevalonate pathway in the cytosol. However, there is significant evidence to suggest intermediate exchange between compartments. In Fig. 4, the initial decline in the concentration of 3-carene at 50 h coincides with an increase in the signal at *m/z* 205, the protonated sesquiterpene parent ion. At the onset of ripeness, sesquiterpenes were the dominant species, the most abundant of which was found to be germacrene D. The sesquiterpene signal reached a peak at around 250 h into the experiment before declining slightly. Previous studies have observed the skin specific occurrence of germacrene D on the ‘Kensington Pride’ cultivar (Lalel et al. 2003).

As can be seen in Fig. 3, germacrene D was the most abundant terpene measured by GC–MS, contributing over 93 % of the total sesquiterpene peak area, other sesquiterpenes identified by GC–MS were analysed at ≤1 % total sesquiterpene peak area including bicyclosesquiphellandrene, copaene, α- and β-cubebene, β-caryophyllene, α-gurjinene, α-Ylangene, γ- and λ-cadinene, λ- and α-muurolene and α-cedrene. MacLeod and Snyder reported a low ratio of mono to sesqui-terpenes (presumably following VOC analysis at an early stage of the ripening process). Li et al. (2009) demonstrated the prediction of ‘Tommy Atkins’ ripening and rot using an electronic nose technique, noting a result similar to Lalel et al. (2003) whereby CO_2_ reached a peak on the 4th day of ripening which coincided with a rise in signal from their most predictive enose peak. Based on previous work on ‘Kensington Pride’ mangoes they attributed this peak to α-terpinolene, our study reveals that the identity of this peak was most likely a sesquiterpene compound.

#### Methanol (m/z 33)

Following the rise in germacrene D (*m/z* = 205; see Fig. 4), methanol (*m/z* = 33) increased resulting in peak levels at around 270 h before declining sharply. Methanol production is likely a result of pectin degradation by pectin methyl esterase (PME). PME catalyses the de-esterification of methylated pectin forming methanol. The de-esterified pectin is then susceptible to hydrolysis by polygalacturonase which breaks down the cell walls and softens the mango tissue. Increased polygalacturonase activity has been observed at the climacteric stage in mango. Roe and Bruemmer (1981) found that a maximum loss in firmness in climacteric fruits could be correlated with a rapid increase in polygalacturonase activity. However, the slow ripening of ‘Abu-Samaka’ mango observed by Abu-Sarra and Abu-Goukh (1992) during elevated polygalacturonase activity, shows that PME plays a key role in the softening of ripening mango as has been observed in other fruits e.g., tomato (Hobson 1965).

#### Acetaldehyde and ethanol (m/z 45, 47 and 29)

Following fruit softening, anaerobic respiration leads to an increased concentration of acetaldehyde: acetaldehyde emissions rose rapidly after 200 h and peaked at around 300 h, declining significantly after that. Similarly, the anaerobic metabolite ethanol rises in concentration following acetaldehyde with an offset of over 50 h. Anaerobiosis within the cytoplasm of mature mango cells is likely prompted by cell membrane damage. Acetaldehyde and ethanol are largely controlled by pyruvate decarboxylase and alcohol dehydrogenase (ADH). ADH is an oxidoreductase which reversibly converts acetaldehyde to ethanol under anaerobic conditions and has been implicated as a stress response for fruits. Furthermore, acetaldehyde is the precursor, via acetyl Coenzyme A, of mevalonic acid (the precursor of monoterpenes and sesquiterpenes. At the final stage of fruit ripening, the monoterpene signal at *m/z* = 137 rises once more and it is interesting to note that in oranges, for example, a rise in acetaldehyde concentrations at fruit maturity has been linked with an increase in limonene production (Pesis and Avissar 1988).

#### Esters (m/z 89, 103, 117, 131, 145, 159, 173, 201, 229 and 257)

The final important class of compounds emitted by the mangoes at the fully ripe stage (after 300 h) was the esters. The esters completely dominate the spectra at the overripe stage, although some of the heavier esters (C_10_ and C_8_) showed signs of peaking after around 400 h of analysis. Significant ester emissions became apparent after 200 h and rose exponentially. Esters are synthesized in vivo from organic acids and alcohols. Catabolysis of fatty acids, potentially producing aroma compounds proceeds via either β-oxidation or by the lipoxygenase pathway. β-oxidation may be the main pathway producing aroma compounds from fatty acids in intact fruit, whereas the lipoxygenase pathway may be the main pathway producing volatile aroma compound in disrupted tissues. Oxidation of fatty acids generates alcohols and acyl Coenzyme A; the precursors to ester formation. Acyl Coenzyme A reductase catalyses reduction to aldehydes which are then converted to alcohols by ADH. Esters are formed by the action of alcohol acyltransferase enzymes on alcohols. Alcohol acyltransferases have been linked to fruit ripening and ethene production in other fruits (Perez et al. 1996). The biosynthesis of fatty acids has previously been observed during the ripening of mango (Bandyopadhyay and Gholap 1973). Large amounts of ethyl butanoate have previously been observed in ‘Kensington Pride’, ‘Baladi’ and Cuban cultivars (Engel and Tressl 1983; Bartley and Schwede 1987; Pino et al. 1989). Lalel et al. (2003) noted that ester production increased exponentially as fatty acid levels increased (also noting the possibility of de novo synthesis) and that the most abundant ester at the fully ripe stage was ethyl octanoate. Both’Keitt’ and’Tommy Atkins’ followed the ester emission pattern whereby ethyl acetate (the most abundant ester signal) rose before the next most abundant ester signal, the C_6_ ester, increased. The ethyl acetate and C_6_ ester signals continued to increase to the end of the analysis. In contrast, the third and fourth most abundant ester ions (the C_8_ and C_10_ esters) reached a maximum and plateau at approximately 350 h. Finally, the next two most abundant ester ions were the C_5_ and C_7_ esters, which continued to rise to the end of analysis.

## Conclusions: can headspace VOC emissions be used to determine mango ripeness?

By observing in real-time the chemodynamics of volatile compound production, emission patterns have been elucidated for the maturation process. Measurements by PTR–ToF–MS produced an information rich dataset, from which several key mass channels were identified and speciated based on a PCA model in conjunction with analysis by ATD–GC–MS. By relating individual ion signal profiles (proportional to concentration profiles) obtained by PTR–ToF–MS to the biochemical pathways that produce volatile organic species, several ions were selected as metabolomic markers of fruit ripeness. Using this approach, the initial complex dataset was reduced to a small number of compounds that not only appear to distinguish ripeness state but also fruit variety.

**Fig. 5:**
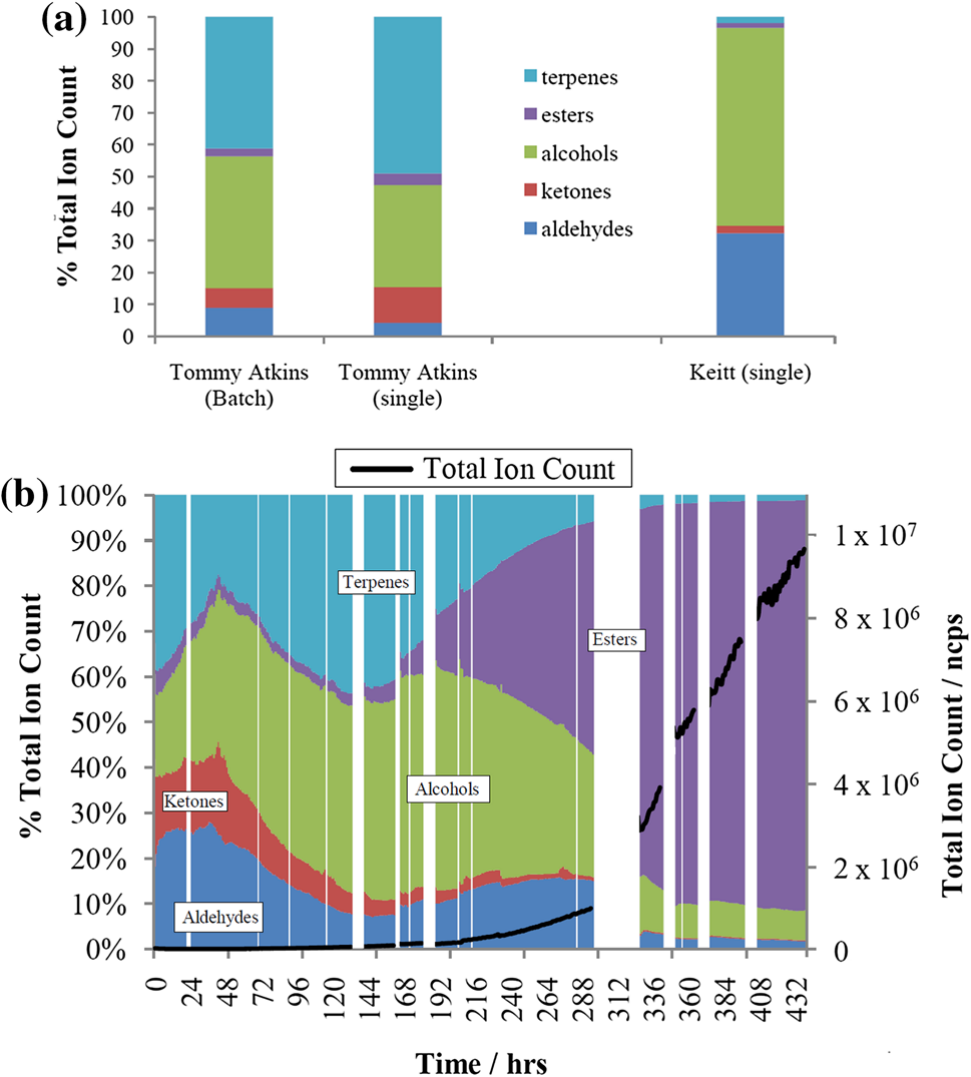
**a** Comparison of relative contribution of different compound classes for stage II ripened mangoes to the total signal of lightweight VOCS (based on ions assignments outlined in this paper (terpenes = 81, 109, 121, 137, 194, 205; alcohols = 29, 33, 47; ketones = 59, 73; aldehydes = 45; esters = 89, 103, 117, 131, 145, 159, 173, 201, 229 and 257). **b** % ion count of lightweight VOCs measured during the ‘Tommy Atkins’ batch experiment based on ions assignments outlined in this paper (terpenes = 81, 109, 121, 137, 194, 205; alcohols = 29, 33, 47; ketones = 59, 73; aldehydes = 45; esters = 89, 103, 117, 131, 145, 159, 173, 201, 229 and 257)

Based on this rationale, the aforementioned ions selected to represent mono- and sesquiterpenes, methanol, ethanol, acetaldehyde, and esters were by grouped by compound class (alongside ions 59 and 73, chosen to represent ketones) and plotted in Fig. 5. Figure 5a shows how the proportion of different compound classes varied between ripe ‘Tommy Atkins’ and ‘Keitt’ mangoes. Whilst a small difference can be seen when comparing the results from the ‘single’ mango to ‘batch’ mango experiments, the proportion of compounds emitted from ‘Keitt’ mangoes clearly forms a very different pattern. One of the main differences between the compounds analysed in ripe ‘Keitt’ mango headspace was the absence of a strong sesquiterpene signal when compared to ‘Tommy Atkins’ [consistent with MacLeod and Snyder’s observations for Keitt mangoes (MacLeod and Snyder 1985)].

Figure 5b shows how the proportion of these compounds varied over the ‘batch’ ‘Tommy Atkins’ experiment. The relative proportion of esters to terpenes in Fig. 5 compares well with previously reported measurements of flavour compounds in mango (MacLeod and Snyder 1985). However, the percentage of alcohols, ketones and aldehydes is quite different possibly owing to the sensitivity of PTR–ToF–MS to lightweight oxygenated VOCs, often previously unreported where headspace measurements of aroma volatiles have been made probably due to their common use as solvents.

Isomers cannot be distinguished by PTR–ToF–MS, furthermore, fragmentation of unidentified compounds in the sample mixture render peak assignment tentative. However, it is not within the scope of this study to provide a comprehensive list of all volatile compounds emitted during ripening (less-volatile compounds may be undetectable using this technique and compounds may have been emitted below the limit of detection associated with PTR ionization). However, in using this technique, it may be possible to define a quantitative estimate of fruit ripeness by examining the relative abundance of different species within PTR mass spectra. For example, based on the results presented in Fig. 5b, a period of fruit ripeness can be defined where sesquiterpenes dominate the total hydrocarbon signal at around 100 h of analysis. This period extends to approximately 300 h to the point where the methanol concentration peaks and before the large rise in ester concentration. Figure 5b exemplifies how this approach can be used to obtain a snapshot of the ripening process, which could ultimately be applied to rapidly profile fruit of different varieties at different ripeness stages.

This work demonstrates the potential of PTR–ToF–MS as a real-time, non invasive detector for use in fruit ripening studies and fruit quality determination. These results are consistent with the fruit quality studies of Taiti et al. (2015) whereby significant differences were consistently recorded for several mass channels between headspace samples of fruit that had been transported via different shipping systems. Techniques such as these are needed in order to assess handling and storage strategies. In addition, this technique could be used to obtain the background information required to utilize other headspace analysers and electronic nose technologies, supporting the results obtained by, for example, Li et al. (2009). Techniques such as the zNose used by Li et al. are based upon pattern recognition; the results from this study suggest that the headspace emission pattern during the fruit ripening process changes markedly. If the information gained from studies such as these can be applied to small, hand-held electronic noses, potentially they could be deployed to assess fruit maturity prior to picking and thus determine the optimum point to harvest mature, green mangoes.
